# Acute Cholecystitis in an Elderly Patient With Antineutrophil Cytoplasmic Antibody-Associated Vasculitis: A Case Report

**DOI:** 10.7759/cureus.21877

**Published:** 2022-02-03

**Authors:** Ryuichi Ohta, Hirotaka Ikeda, Saya Kubota, Chiaki Sano

**Affiliations:** 1 Community Care, Unnan City Hospital, Unnan, JPN; 2 Hematology, Japanese Red Cross Society Himeji Hospital, Hyogo, JPN; 3 Community Medicine Management, Shimane University Faculty of Medicine, Izumo, JPN

**Keywords:** rural hospitals, cholecystectomy, exploratory laparoscopy, elderly, acute cholecystitis, anca-related vasculitis

## Abstract

A diagnosis of antineutrophil cytoplasmic antibody (ANCA)-associated vasculitis is difficult to establish in elderly patients. Herein, we report a case of acute cholecystitis mimicking sepsis in an elderly patient with ANCA-associated vasculitis. A 99-year-old woman was transferred to a rural community hospital on account of anorexia and hypotension; there, she was initially diagnosed with sepsis and treated accordingly. However, she developed new-onset right upper quadrant tenderness on indirect fist percussion of the liver, and Murphy’s sign was positive. While imaging did not reveal any findings suggestive of cholecystitis, the high index of suspicion for cholecystitis prompted an exploratory laparoscopy. Intraoperatively, the gallbladder wall was found to be inflamed, necessitating laparoscopic cholecystectomy. Histopathologic examination of the resected gallbladder showed neutrophilic infiltration and fibrinoid necrosis of the arterial walls. Perinuclear ANCA titers were elevated. These findings were consistent with a diagnosis of ANCA-associated vasculitis, and treatment with prednisolone markedly improved her condition. This case shows the difficulty encountered in differentiating between sepsis and ANCA-related vasculitis based on clinical features and relatively non-invasive diagnostic strategies alone. This study highlights the utility of invasive diagnostic procedures (e.g., biopsy) in elderly patients in whom a diagnosis of ANCA-associated vasculitis is difficult to establish.

## Introduction

Antineutrophil cytoplasmic antibody (ANCA)-associated vasculitis is an autoimmune disease that commonly affects the skin, kidneys, lungs, and nerves [1] and presents as a rash, nephritis, pulmonary infiltration, and peripheral neuropathy [2]. Histopathologic analysis of biopsy specimens is crucial in establishing a diagnosis of ANCA-related vasculitis [3], and diagnosis can be challenging in cases where a biopsy cannot be performed. Moreover, various diseases presenting with multiorgan damage (e.g., sepsis) can mimic ANCA-associated vasculitis [4]; therefore, these differential diagnoses should be ruled out before making a diagnosis of ANCA-associated vasculitis.

The diagnosis of ANCA-related vasculitis is even more difficult in older patients owing to the various comorbidities they may have that can complicate the history and physical examination findings [5] and alter the clinical course of sepsis from acute to subacute, similar to ANCA-associated vasculitis [6]. Furthermore, owing to their immunocompromised state, elderly individuals are at a higher risk of developing sepsis [7]. While the biopsy is essential to confirm a diagnosis of ANCA-associated vasculitis, its utility in older patients is limited due to its invasiveness.

To the best of our knowledge, this is the first report of an elderly patient with cholecystitis who was pathologically diagnosed with ANCA-related vasculitis. Herein, we report a case of acute cholecystitis mimicking sepsis in an elderly patient with ANCA-associated vasculitis.

## Case presentation

A 99-year-old woman presented with anorexia and new-onset hypotension following a one-week history of fever and cough (which was left untreated), prompting admission to a rural community hospital. Her past medical history revealed hypertension, controlled with nifedipine, and osteoporosis. Prior to admission, the patient was independent in performing activities of daily living.

Evaluation of her vital signs revealed a slightly elevated temperature (37.7 °C) and reduced blood pressure (96/62 mmHg) and oxygen saturation (SpO2; 92% at room air). Heart (82 bpm) and respiratory (20 bpm) rates were normal. Head and neck examination showed no cervical lymphadenopathies. Chest auscultation revealed bilateral generalized crackles. Her abdomen was soft and non-tender. Examination of the knees and hands showed no evidence of arthritis.

Laboratory results on admission are presented in Table 1 and were notable for anemia, leukocytosis, and elevated C-reactive protein levels.

**Table 1:** Laboratory data on admission and Day 7 of hospitalization PT, prothrombin time; INR, international normalized ratio; APTT, activated partial thromboplastin time; UIBC, unsaturated iron-binding capacity; eGFR, estimated glomerular filtration rate; CK, creatine kinase; CRP, C-reactive protein

Laboratory Test	Day 1	Day 7	Reference
White blood cells	12.9 × 10^3^	12.5 × 10^3^	3.5–9.1 × 10^3^/μL
Neutrophils	91.9	93.2	44.0–72.0%
Lymphocytes	3.9	2.8	18.0–59.0%
Monocytes	2.8	3.1	0.0–12.0%
Eosinophils	1.2	0.6	0.0–10.0%
Basophils	0.2	0.3	0.0–3.0%
Red blood cells	2.73 × 10^6^	2.65 × 10^6^	3.76–5.50 × 10^6^/μL
Hemoglobin	7.9	7.5	11.3–15.2 g/dL
Hematocrit	23.8	23.2	33.4–44.9%
Mean corpuscular volume	87.2	87.5	79.0–100.0 fL
Platelets	57.9 × 10^4^	69.0 × 10^4^	13.0–36.9 × 10^4^/μL
Total protein	5.2		6.5–8.3 g/dL
Albumin	1.6	1.3	3.8–5.3 g/dL
Total bilirubin	0.3	0.2	0.2–1.2 mg/dL
Aspartate aminotransferase	19	29	8–38 IU/L
Alanine aminotransferase	11	14	4–43 IU/L
Alkaline phosphatase	244	194	106–322 U/L
γ-Glutamyl transpeptidase	19	23	<48 IU/L
Blood urea nitrogen	17.8	13.7	8–20 mg/dL
Creatinine	0.85	0.60	0.40–1.10 mg/dL
Serum Na	139	138	135–150 mEq/L
Serum K	4.5	4.2	3.5–5.3 mEq/L
Serum Cl	106	104	98–110 mEq/L
CRP	30.1	14.76	<0.30 mg/dL
Procalcitonin	0.25		0–0.05 ng/mL
IgG	1158		870–1700 mg/dL
IgM	40		35–220 mg/dL
IgA	201		110–410 mg/dL
KL-6	313		105.3-401.2 U/mL
SP-D	46.9		<100 ng/mL
SP-A	31.6		<31.6 ng/mL
Leukocyte	(-)	(-)	
Nitrite	(-)	(-)	
Protein	(-)	(-)	
Glucose	(-)	(-)	
Urobilinogen	(-)	(-)	
Bilirubin	(-)	(-)	
Ketone	(-)	(-)	
Blood	(-)	(-)	
pH	7.0	6.0	
Specific gravity	1.017	1.011	

**Figure 1:**
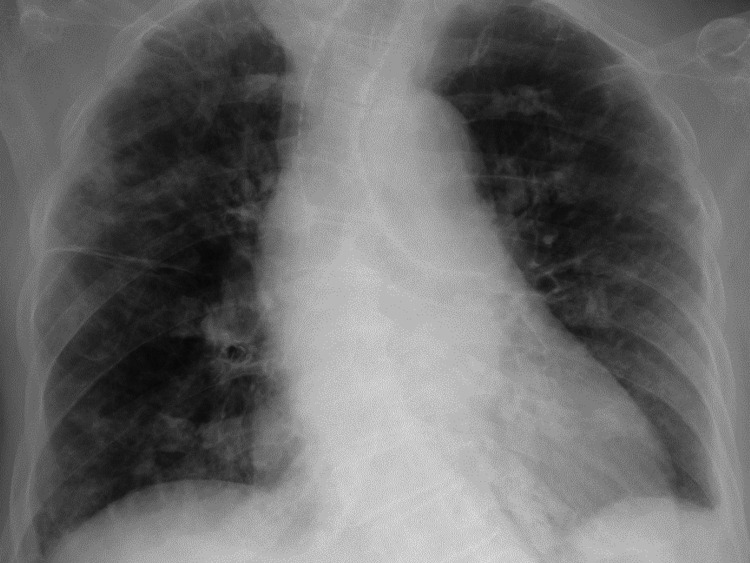
Initial chest radiograph showing bilateral pulmonary infiltrates

**Figure 2:**
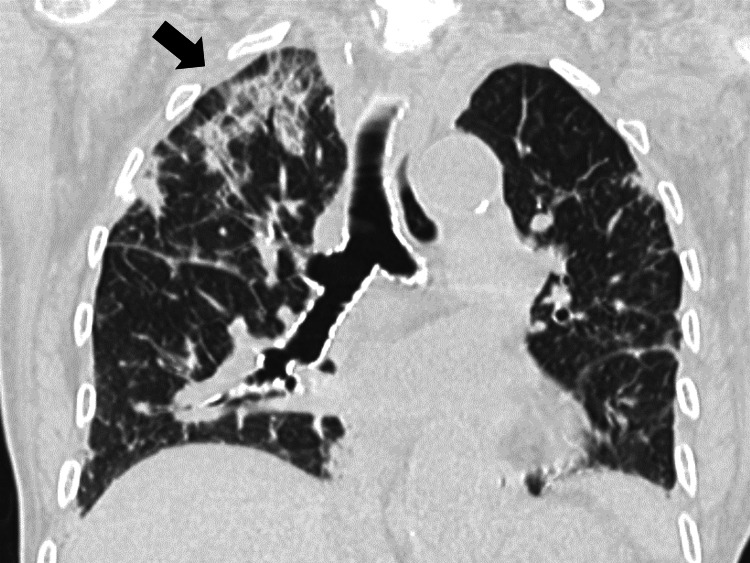
Initial chest computed tomography showing bilateral pulmonary infiltrates predominantly on the right lung

Chest radiography (Figure 1) and computed tomography (CT) (Figure 2) demonstrated bilateral pulmonary infiltrates.

Sputum Gram staining showed neutrophilic and polymicrobial patterns. Regarding anemia, we performed an upper gastroduodenoscopy and a blood stool test, neither of which showed bleeding. We suspected interstitial pneumonia, but KL-6 and other factors indicative of interstitial pneumonia were negative, ruling out the disease. These findings were consistent with a diagnosis of bacterial pneumonia and sepsis, prompting treatment with ceftriaxone (2 g/day) and intravenous fluids. After initiation of treatment, her blood pressure normalized even though vasopressors were not used.

**Figure 3:**
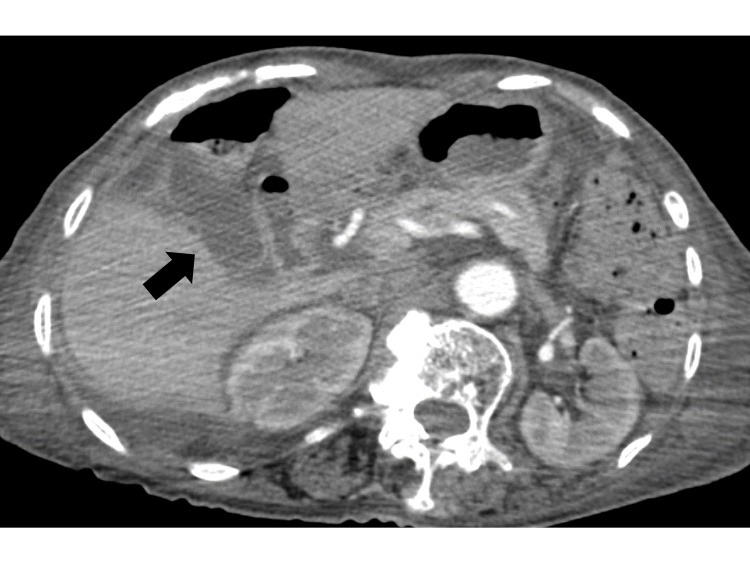
Contrast-enhanced abdominal computed tomography revealing gallbladder edema without strong wall enhancement

On the seventh day of admission, the patient developed right upper quadrant tenderness on indirect fist liver percussion and Murphy’s sign was positive. Contrast-enhanced abdominal CT did not reveal any evidence of cholecystitis or cholangitis (Figure 3).

Liver function tests were unremarkable. Because of the absence of findings suggestive of other inflammatory lesions, we suspected cholecystitis based on the physical examination features.

**Figure 4:**
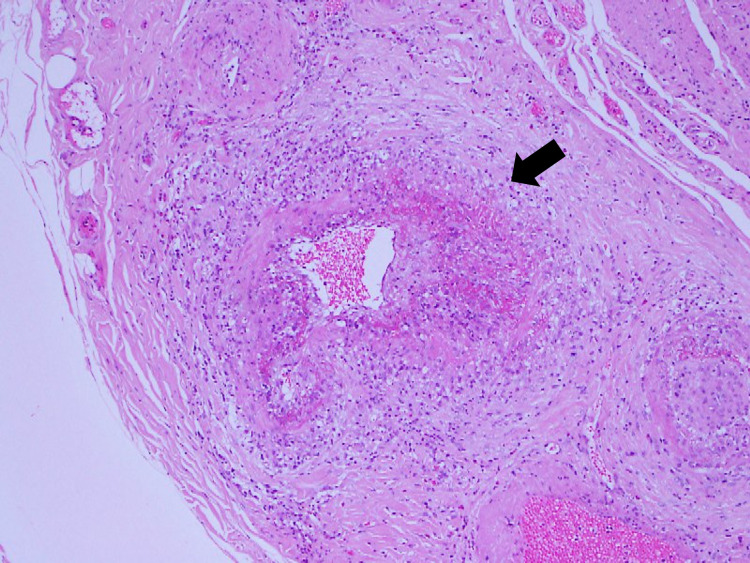
Histopathologic analysis of the gallbladder wall demonstrating neutrophilic infiltration with fibrinoid necrosis on the arterial walls (hematoxylin and eosin stain, ×40)

The patient underwent exploratory laparoscopy, which revealed an inflamed gallbladder wall. Cholecystectomy was then performed, and no organism was detected on gram staining of bile. Histopathologic analysis of the resected gallbladder showed neutrophilic infiltration and fibrinoid necrosis of the arterial walls (Figure 4).

Postoperatively, the abdominal pain resolved but perinuclear antineutrophil cytoplasmic antibodies (p-ANCA) were elevated at 263 U/ml (normal range <3.5 U/ml). These findings were consistent with a diagnosis of ANCA-associated vasculitis with cholecystitis.

The patient was treated with prednisolone (1 mg/kg) and the fever resolved; we gradually tapered the drug. She was placed on a maintenance dose of prednisolone (3 mg/day) for two years, and her blood pressure normalized without nifedipine. Anemia, which could have resulted from vasculitis, resolved as well (hemoglobin, 11.7 g/dL).

## Discussion

This case shows the difficulty encountered in differentiating the history and physical examination findings of sepsis from those of ANCA-associated vasculitis. Even among elderly patients, invasive diagnostic procedures play important roles in the diagnosis and subsequent treatment of ANCA-associated vasculitis.

The differentiation between sepsis and ANCA-associated vasculitis is even more challenging in patients with complicated clinical conditions, requiring close follow-up. Both medical conditions can induce dehydration, leading to shock [8]. Elderly individuals are at higher risk of dehydration even in mild conditions, owing to the physiologic changes associated with aging [9]. Moreover, sepsis and ANCA-associated vasculitis can both cause multiorgan failure due to systemic or arterial inflammation [10]. In most cases, the degree of disease progression may help differentiate the two conditions. However, many diseases, including sepsis, have atypical clinical courses in elderly patients [11], making the diagnosis of ANCA-associated vasculitis difficult. Thus, ANCA-associated vasculitis should be considered in patients being managed for sepsis. Close follow-up of their symptoms is critical to accurately detect vasculitis.

Furthermore, although rare, the coexistence of sepsis and ANCA-associated vasculitis should be considered. In our case, the patient got slightly better with the administration of antibiotics for sepsis and pneumonia, but the fever was persistent due to vasculitis. She might have had a case of coexistence of sepsis and vasculitis. Older patients have weak immune systems, so early treatment of sepsis is vital. For the management of fever in older patients, sepsis should be treated promptly if suspected, the persistence of fever despite treatment should be investigated, and other inflammatory diseases, such as vasculitis, should be ruled out.

The clinical features of cholecystitis secondary to ANCA-associated vasculitis may differ from those of calculous cholecystitis. Calculous cholecystitis refers to the obstruction of the cholecystic duct by gallstones, resulting in an increased intracystic pressure that may cause wall ischemia and infection, affecting all layers of the wall of the gallbladder [12]. In contrast, cholecystitis secondary to ANCA-associated vasculitis is directly caused by gallbladder wall inflammation [13]. Because gallbladder wall inflammation in ANCA-associated vasculitis is most often focal, the typical imaging findings of cholecystitis (e.g., gallbladder dilatation and diffuse wall thickening) are rarely seen [14]. The clinical course of calculous cholecystitis can be acute pain and consecutive fever and sepsis, which may be different from cholecystitis secondary to vasculitis, which manifests as mild abdominal pain and persistent fever of gradual onset [12-13]. As such, this can delay the diagnosis of cholecystitis in patients with ANCA-associated vasculitis.

The use of a multidisciplinary approach in the diagnosis and management of acute cholecystitis in elderly patients with ANCA-associated vasculitis is very important. In this case, the surgeon was initially reluctant to perform invasive interventions on a 99-year-old patient. However, the development of clinical features consistent with cholecystitis (i.e., right upper quadrant tenderness on indirect fist liver percussion and a positive Murphy’s sign) [15] strengthened the decision to push through with invasive interventions, which later revealed ANCA-associated vasculitis. This highlights the importance of going against age-related stereotypes that limit the use of invasive procedures in elderly patients [16].

## Conclusions

We encountered a case of acute cholecystitis mimicking sepsis in an elderly patient with ANCA-associated vasculitis. This study illustrates the difficulty encountered in differentiating ANCA-associated vasculitis from sepsis based on clinical features and relatively non-invasive diagnostic strategies alone and highlights the utility of invasive diagnostic procedures (e.g., biopsy) in elderly patients in whom the diagnosis of ANCA-associated vasculitis is difficult to establish.
